# Lady with seizure and skin lesions

**DOI:** 10.11604/pamj.2020.35.31.21170

**Published:** 2020-02-07

**Authors:** Avik Panigrahi, Abheek Sil

**Affiliations:** 1 Department of Dermatology, Venereology, and Leprosy, RG Kar Medical College, Kolkata, West Bengal, India

**Keywords:** Angiofibromas, ungual fibroma, gingival fibroma, ash-leaf macule, epilepsy, tuberous sclerosis

## Image in medicine

A 35-year-old Indian lady, born out of non-consanguineous parentage, presented with multiple asymptomatic bumps over face and fingers. The facial lesions had started appearing since 2 years of age and finger lesions were first noticed during her adolescence. She had been on anti-epileptic medication for recurrent episodes of seizures since last 15 years. Her family history was insignificant. Examination revealed multiple non-tender, discrete, yellowish-brown to skin-coloured, soft-to-firm papules (1 to 3mm) distributed symmetrically over centrofacial region. Multiple firm, non-tender, skin-coloured nodules over proximal nail fold along with longitudinal nail grooving were noted (Panel A, red arrow). A small firm skin-coloured plaque (Panel B, red arrow) and a leaf-shaped hypopigmented macule (Panel B, black arrow) were observed over thigh. Oral examination revealed a non-tender soft-to-firm papule over upper gingival (Panel A, black arrow). Systemic examination was insignificant. Routine blood investigations, echocardiography, ultrasonography of abdomen and fundoscopy of eyes failed to reveal any abnormality. Magnetic resonance imaging of the brain revealed cortical tubers and calcified subependymal nodules.

**Figure 1:**
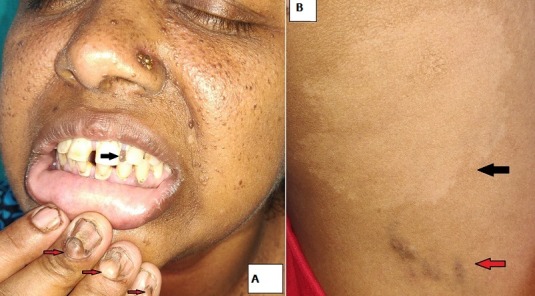
(A) Multiple discrete, yellowish-brown to skin-coloured, soft-to-firm papules distributed over centrofacial region (facial angiofibromas), (red arrow) skin coloured papules over proximal nail fold (ungual fibroma) along with longitudinal nail grooving, (black arrow) soft-to-firm nodule over upper gingiva (gingival fibroma); (B (red arrow) a firm, non-tender, skin coloured plaque (shagreen patches) and (black arrow) a leaf-shaped hypopigmented macule over thigh (ash-leaf macules)

